# Mechanical, Thermal, and Shape Memory Properties of Three-Dimensional Printing Biomass Composites

**DOI:** 10.3390/polym10111234

**Published:** 2018-11-07

**Authors:** Hongjie Bi, Min Xu, Gaoyuan Ye, Rui Guo, Liping Cai, Zechun Ren

**Affiliations:** 1 Key Laboratory of Bio-Based Material Science and Technology (Ministry of Education), Material Science and Engineering College, Northeast Forestry University, Harbin 150040, China; bihongjie1016@163.com (H.B.); 15764226279@163.com (G.Y.); guorui0527@163.com (R.G.); yourong_rzc@163.com (Z.R.); 2 Mechanical and Energy Engineering Department, University of North Texas, Denton, TX 76201, USA; liping.cai@unt.edu; 3 College of Materials Science and Engineering, Nanjing Forestry University, Nanjing 210037, China

**Keywords:** wood flour/thermoplastic polyurethane blend, EPDM-*g*-MAH, interface bonding, shape memory

## Abstract

In this study, a series of heat-induced shape memory composites was prepared by the hot-melt extrusion and three-dimensional (3D) printing of thermoplastic polyurethane (TPU) using wood flour (WF) with different contents of EPDM-*g*-MAH. The mechanical properties, microtopography, thermal property analysis, and heat-induced shape memory properties of the composites were examined. The results showed that, when the EPDM-*g*-MAH content was 4%, the tensile elongation and tensile strength of the composites reached the maximum value. The scanning electron microscopy and dynamic mechanical analysis results revealed a good interface bonding between TPU and WF when the EPDM-*g*-MAH content was 4%. The thermogravimetric analysis indicated that the thermal stability of TPU/WF composites was enhanced by the addition of 4% EPDM-*g*-MAH. Heat-induced shape memory test results showed that the shape memory performance of composites with 4% EPDM-*g*-MAH was better than that of unmodified-composites. The composites’ shape recovery performance at a temperature of 60 °C was higher than that of the composites at ambient temperature. It was also found that, when the filling angle of the specimen was 45°, the recovery angle of the composites was larger.

## 1. Introduction

Shape memory polymers (SMPs) are an important type of stimuli-responsive material that have the ability to be fixed to a temporary shape and then recover their original shapes in response to an external stimulus [1,2,3]. SMPs can be widely applied in intelligent material, aerospace structures, biomedical devices, piezoresistive sensors, and electronic apparatuses [4,5,6,7]. On the basis of elastomers, the polymer essentially shows excellent shape memory effects and has the advantages of a large recovery ability, flexible transition temperatures, a light weight, and a superior processing ability [4,5,6,8]. Thermoplastic polyurethane elastomer (TPU) is a soft material with flexibility, durability, and good entropic elasticity, and has an excellent shape memory function [9,10]. TPU is a kind of block copolymer composed of soft and hard segments, which presents a series of characteristics, depending on the ratio of hard and soft segments [11]. The soft segments are composed of polyester or polyether, and the hard segments are usually composed of diisocyanate and a benzyl structure [12]. Soft segments serve as switches to maintain temporary shapes, while the hard segments are used to memorise initial shapes [13]. The polyurethane series matrix composites have been widely studied [8,9,14], such as the polyurethane(PU)/cellulose nanocrystals nanocomposites, cellulose nanowhiskers (CNWs)-PU composites [6], poly (lactic acid) (PLA)/TPU-cellulose nanofiber nanocomposites [10], TPU/poly (ε-caprolactone) (PCL) blends, poly(vinyl alcohol) particle-TPU composites [15], and poly (glycerol sebacate urethane)-cellulose nanocomposites [16].

In recent years, such kinds of shape memory composites have attracted great attention due to their function of active-shape changing [2,9,16]. However, the major drawback of the material is that it is difficult to control the sizes and geometries of shape memory structures using traditional fabrication techniques [2,17]. 3D printing would provide a facile approach to achieving complex structures of materials, producing cheaper, faster architectures with specially designed sizes and geometries [17,18,19,20]. In this study, we focused on fused deposition modeling (FDM) of 3D printing. In the FDM process, the material was fed to the heated nozzles in the form of a filament, which was melted at the nozzles, extruded, and deposited on the printing platform, and a 3D solid structure was formed layer by layer [19,21,22,23,24]. With the advancements of 3D printing and the rapid developments in smart materials, many studies have been implemented for smart materials based on 3D printing shape memory architectures [2,9,22,25]. Wei et al. realized 4D active shape-changing structures based on the SMPs and direct-write fabrication [2]. Monzón et al. controlled the property of the programmed shape by controlling temperature, and showed that the property of the shape memory of the 3D printed parts had been retained and could be utilized as a mechanical actuator [9]. Nadgorny et al. proposed pH-responsive polymers with an affordable 3D Printer [22].

In this study, the TPU composite filaments were used in combination with an FDM 3D printer to fabricate the structure of biomass composites with a shape memory performance. However, a major drawback of TPU matrix composites is their high cost, which may limit their applications. Wood flour (WF) is an abundant biomass material that is friendly to the environment, low cost, and low density, and the effect of different contents of WF on the performance of TPU composites was discussed in our previous research [26]. Using EPDM-*g*-MAH as a modifier can compensate for the toughness loss and improve the interfacial adhesion of the TPU/WF [26]. However, few studies have shown that the appropriate amount of added EPDM-*g*-MAH is suitable. In this study, the composites were made by adding 20% WF into TPU. As far as we know, few researchers have added such a large amount of biomass fiber to composites, which undoubtedly reduces costs. The composite’s optimum ratio was optimized by adding different contents of EPDM-*g*-MAH, and the effect of different contents of EPDM-*g*-MAH on the properties of composites was explored. Then, the heat-induced shape memory process and shape memory effect mechanism of TPU/WF composites were investigated under different experimental conditions.

Firstly, the effects of different contents of EPDM-*g*-MAH on the tensile properties of WF/TPU composites were explored. Then, the dynamic mechanical properties, microtopography, and thermal properties of composites were investigated. In addition, the shape memory properties of the printed object were analyzed. Finally, the effect of the program temperature of the specimen and the filling angle in the 3D printing process on the shape memory performance were discussed in detail.

## 2. Materials and Methods

### 2.1. Materials

The TPU (Elastollan C85A) and EPDM-*g*-MAH were obtained from Deansheng Plastic company (Dongguan, China). The poplar wood flour with an average diameter of 150 μm was supplied by Lingshou County Mineral Processing plant (Shijiazhuang, China). Before any processing, the TPU and WF were dried in an oven at 103 °C for at least 12 h and EPDM-*g*-MAH was dried at 80 °C for 6 h.

### 2.2. Composites Preparation

TPU and WF blends with varying amounts of EPDM-*g*-MAH loading (2–10 wt %) were prepared by the melt processing technique. The blends (80:20 wt % of TPU:WF) filled with EPDM-*g*-MAH were weighed, and then blended for 10 min using a high-speed mixer (SHR-10A, Zhangjiagang Tongsha Plastic Machinery Company, Zhangjiagang, China) at room temperature. The mixture was poured into the twin-screw extruder (SJSH30/SJ45, Nanjing Xiangsu Machinery Factory, Nanjing, China) at 195 °C (die temperature) with a screw speed of 20 RPM, followed by natural cooling, and used a grinder (PC-180A, Longhe Plastics Machinery Company, Chaozhou, China) to make composite particles. The average diameter of the composite particles was 4 mm. The prepared composites were designated as x%-TPU/WF, in which, the EPDM-*g*-MAH content was x%, designated as 2%-TPU/WF, 4%-TPU/WF, 6%-TPU/WF, 8%-TPU/WF, and 10%-TPU/WF, respectively. The composite particles were added into a single-screw extruder (SHSJ25, Dongguan Songhu Molding Machine Company, Dongguan, China) at 190 °C with a screw speed of 25 RPM, screw L/D of 12:1, and die size of 3.5 mm. Then, the composite filaments with a diameter range of 1.75 ± 0.1 mm were obtained by an extruder and stretched under traction, followed by natural cooling for 24 h. Finally, the specimens were printed by an MR300 3D Printer with different composite filaments. The 100% TPU (85A TPU) was too soft and not able to be FDM-printed as samples for various performance tests using our current FDM 3D printer. Therefore, the study did not process the characterization comparison of 100% TPU. The printing parameters for the FDM printer are listed in Table 1.

### 2.3. Tensile Tests

The tensile properties of the samples were measured at room temperature using an electronic universal mechanical testing machine (RGT-20A, Shenzhen Regal Instrument Company, Shenzhen, China) according to the DIN 53504 test method. The sizes of a dumbbell-shaped specimen were 75 mm × 12.5 mm × 2 mm (*L* × *W* × *T*) and the test was performed with a speed of 500 mm/min at room temperature, and the gauge length was 20 mm. The reported values of each composite were calculated as the averages of eight specimens.

### 2.4. Scanning Electron Microscopy (SEM)

SEM images were observed with the field emission scanning electron microscope (JSM-7500, JEOL, Tokyo, Japan). The TPU/WF composites samples were fractured in liquid nitrogen, sputter-coated with gold, and then examined with SEM. The SEM images were taken from multiple points of the treated specimens.

### 2.5. Dynamic Mechanical Analysis (DMA)

DMA of the samples was conducted in a tensile mode utilizing a dynamic mechanical analyzer (DMA 242C, Netzsch, Bavaria, Germany). Samples for DMA were printed with composite filaments in a rectangular shape (30 mm × 5 mm × 2 mm) and the properties were measured with a temperature sweep of 5 °C/min and a frequency of 1 Hz over a temperature range of −100 to 100 °C.

### 2.6. Thermogravimetric Analysis (TGA)

The thermal stability of the TPU/WF composites was measured using a TG 209 analyzer (Netzsch Instruments, Bavaria, Germany). Approximately 5 mg samples were weighed and then placed in an aluminum pan. Then, the samples were heated at a heating rate of 10 °C/min from 25 to 600 °C under N_2_ atmosphere.

### 2.7. Differential Scanning Calorimetry (DSC)

The thermal performances were evaluated using a DSC 214 analyzer (Netzsch Instruments, Bavaria, Germany) under N_2_ atmosphere. About 5 mg samples were weighed and then placed in an aluminum pan. To eliminate thermal history and remove residual moisture, the first scan of the specimen was heated from −25 up to 230 °C and remained at 230 °C for 5 min. The samples were cooled to −25 °C and then reheated to 230 °C to obtain the fusion point of composites. All scans were performed at the same heating rate of 10 °C/min.

### 2.8. Shape Memory Effect Characterization

As a rectangular strip with a size of 80 mm × 8 mm × 1.2 mm, the specimens for the shape memory behavior test were printed by the FDM printer. The shape memory behaviors of different specimens were compared under different temperature conditions (ambient temperature or 60 °C in oven). The effect of filling angle on the shape memory properties of composites was studied. The 3D printed samples with an original shape (*S*_o_) with an initial angle (*A*_o_ = 180°) were heated in a temperature above the soft segments melt temperature of TPU (*T*_m1_) for 2 h. Secondly, the samples were bent into a temporary shape (*S*_t_) with a fixed angle (A_t_) and placed in a temperature below glass transition temperature (*T*_g_) for 12 h to fix the temporary shape. Finally, the shape recovery process was monitored by placing the temporary samples at the corresponding temperature. By measuring the angle between the straight ends of the bending specimen, the shape recovery angle was determined and the value was directly read from the protractor. Additionally, the recovery angle (*A*_r_) of the samples recovery shape (*S*_r_) was recorded. The shape recovery ratio (*R*_r_) was defined as follows:

R_r_% = (A_r_ − A_t_)/(180 − A_t_) × 100% (1)

The results represented the averages of at least three repeated specimens. The shape recovery demonstrations of the TPU/WF composite specimens were recorded by a video camera.

## 3. Results and Discussion

### 3.1. Mechanical Properties

**Figure 1:**
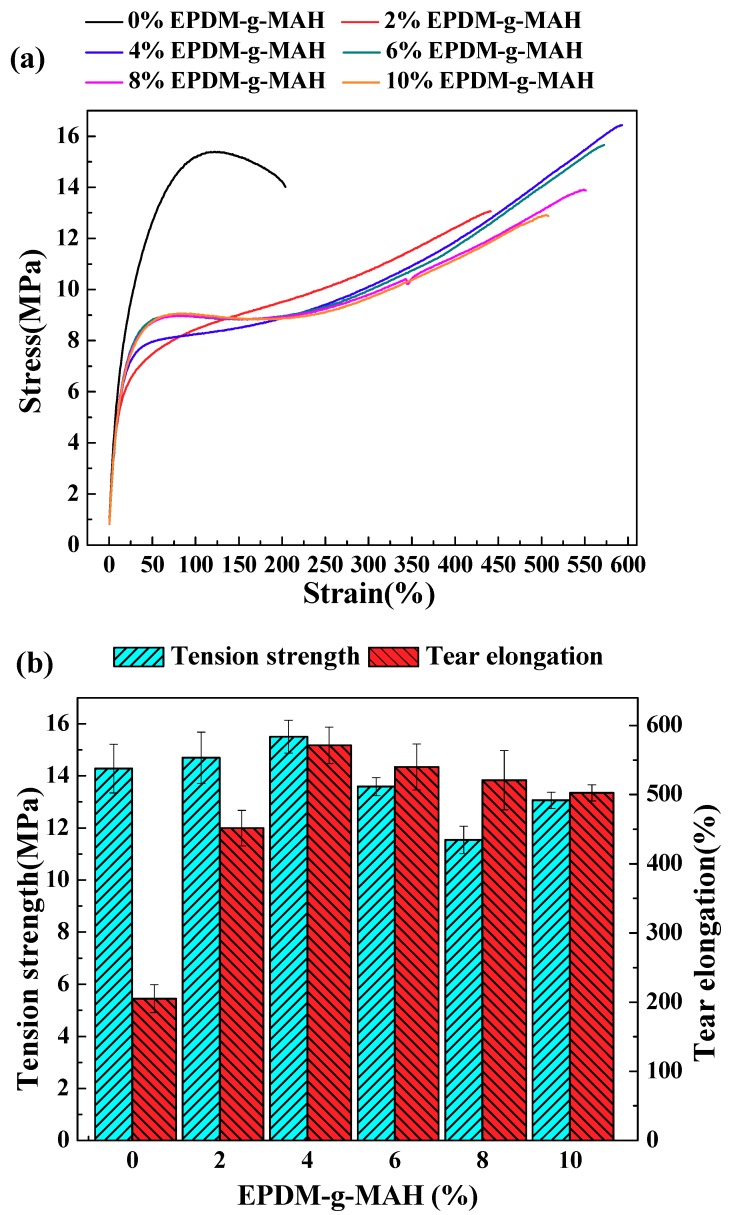
Tensile stress-strain curves (**a**) and tensile strength and tear elongation (**b**) of TPU/WF composites with different EPDM-*g*-MAH loading values.

Figure 1a shows the stress-strain curves of the TPU/WF composites modified with different contents of EPDM-*g*-MAH. Compared to the unmodified-TPU/WF composites, the EPDM-*g*-MAH modified TPU/WF composites showed a significant increase in stress related with the increasing strain. The results of tensile properties were summarized and are presented in Figure 1b. It can be seen that the tear elongation of the TPU/WF composites was enhanced effectively, and reached a maximum for the samples modified by 4% EPDM-*g*-MAH. The tear elongation increased from 205.26% (unmodified-TPU/WF) to 591.17% for 4%-TPU/WF, achieving a 2.9-fold improvement. The ANOVA test indicated that, compared to the unmodified samples, the increase of tear elongation was significant at the α = 0.001 level (*P*-value = 2.4 × 10^−10^). This greatly improved the toughness of composites. The improvements in tear elongating may be attributed to the high interfacial adhesion supplied by the esterification reaction between EPDM-*g*-MAH and WF [26]. When comparing the tensile strengths of the unmodified samples and samples of 4%-TPU/WF, although the large errors are shown in Figure 1b, the ANOVA test showed that the increase of tensile strength was significant at the α = 0.05 level (*P*-value = 0.048). The physical crosslinking between TPU and EPDM can also promote the interface bonding of composites. Therefore, the stress can be transferred effectively from WF to the TPU matrix, and the tensile properties of the composite can be improved.

However, when the EPDM-*g*-MAH content was higher than 4%, both the tensile strength and tear elongation were decreased. When comparing the tensile strengths and tear elongations of the composites between 4%-EPDM-*g*-MAH and 8%-EPDM-*g*-MAH, the ANOVA test showed that the decreases were significant at the α = 0.001 level (*P*-value = 4.01 × 10^−6^) and α = 0.001 level (*P*-value = 0.0017), respectively. The appropriate content of the modifier is related to its coverage on the surface of WF particles. If the content of the modifier is very low, it is difficult to form a good interface to achieve the ideal effect due to the modifier making incompletel contact with the WF particle surface. With the increase of EPDM-*g*-MAH loading, more MAH was contained in the EPDM. When being mixed with WF and TPU, the more severe reaction between the MAH and WF partially limited the chains motion of EPDM and the deformation of TPU particles, resulting in the failure to effectively induce the matrix to generate plastic flow for improving the properties of the blend system [27,28,29]. Therefore, the tear elongation decreased, but it was still better than that of the unmodified composites. The addition of the elastomer EPDM-*g*-MAH decreased the tensile strength of the composites, which was consistent with the results of elastomer-toughened plastics [28]. Moreover, when the EPDM-*g*-MAH content was 4%, the composites had the highest tear elongation, which benefited the shape memory recovery of composites [3]. Therefore, the optimum content of EPDM-*g*-MAH was 4% in the composites.

### 3.2. Morphology of the TPU/WF Composites

**Figure 2:**
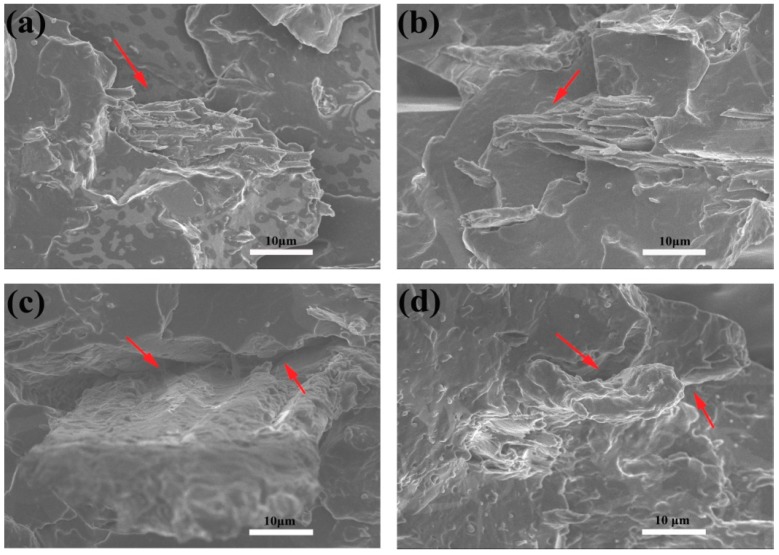
The cross-sectional SEM images showing the microstructure of TPU/WF composites: (**a**) unmodified-TPU/WF composites; (**b**) 4%-TPU/WF composites; (**c**) 6%-TPU/WF composites; (**d**) 8%-TPU/WF composites.

The morphology of the unmodified-TPU/WF and different contents of EPDM-*g*-MAH-modified TPU/WF composites were examined by SEM. Figure 2a shows that the interface of the unmodified-TPU/WF had a few cracks (as the red arrow shown in Figure 2a), and the interphase was clear, indicating that the interface adhesion between TPU and WF was poor. Figure 2b shows that there was a good adhesion as no fiber pullouts or gaps appeared in the 4%-TPU/WF composites, and the interface between WF and TPU became blurry (as the red arrow shown in Figure 2b) [29]. With an increase of EPDM-*g*-MAH content, WF was uniformly dispersed in the TPU matrix and some small aggregations were observed (as red arrows shown in Figure 2c,d), as shown in Figure 2c,d. This phenomenon led to quite poor tensile properties, as the poor interaction would affect the transfer of load to WF. The results stated clearly that, when the EPDM-*g*-MAH content was 4%, the compatibility of WF and TPU could be improved by EPDM-*g*-MAH. Therefore, the content of the EPDM-*g*-MAH played a crucial role in the improvement of the interfacial bonding between the WF and the TPU matrix. This phenomenon is in accordance with the results of the tensile property test.

### 3.3. Dynamic Mechanical Analysis

**Figure 3:**
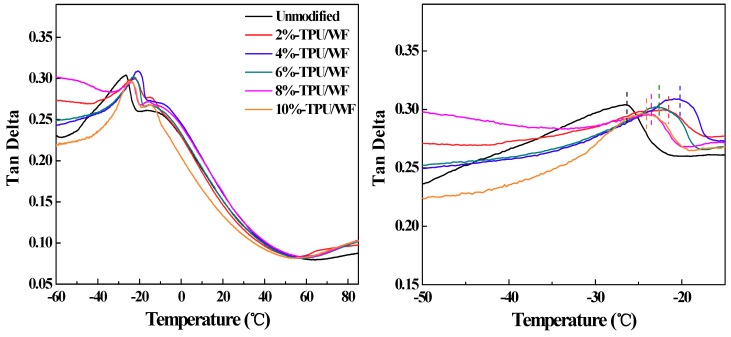
The tan delta (**left**) and a zoomed in view of the peak area (**right**) of TPU/WF composites with different contents of EPDM-*g*-MAH.

Dynamic mechanical analysis was performed on unmodified-TPU/WF, 2%-TPU/WF, 4%-TPU/WF, 6%-TPU/WF, 8%-TPU/WF, and 10%-TPU/WF, respectively. The *T*_g_ was obtained from the peak temperature of tan delta (tan δ) in the curves shown in Figure 3. It can be seen that the *T*_g_ values of the EPDM-*g*-MAH-modified composite were all higher than those of unmodified-TPU/WF. Additionally, the growth trend of *T*_g_ with EPDM-*g*-MAH-modified composites could be explicated by two aspects. On the one hand, with an EPDM-*g*-MAH added, a high cross-linking degree within TPU and the EPDM of EPDM-*g*-MAH resulted in a limitation of the mobility of TPU and WF chains [1,30]. On the other hand, the reaction of the anhydride groups on EPDM-*g*-MAH and the –OH groups on WF caused stronger interfacial interactions between TPU and WF.

It can be seen in Figure 3 that the *T*_g_ of the 4%-TPU/WF composites was the highest one. When the content of EPDM-*g*-MAH was more than 4%, the *T*_g_ of the composites decreased. This may be because when the content of the EPDM-*g*-MAH content was 4%, the contact between the modifier and the WF was the most complete, forming a good stress transfer. When the EPDM-*g*-MAH content was higher than 4%, the reaction between MAH and WF was more intensive, and a large number of WF concentrated in TPU/WF composites, which can cripple the interfacial interactions between TPU and WF, thus reducing the restriction on the mobility of molecular chains [16,28]. This phenomenon was in agreement with the aforementioned tensile and SEM properties. In this study, the cooling temperature of the shape memory test was set to −25 °C, which was below the *T*_g_ of the prepared shape memory TPU/WF composites.

### 3.4. Thermal Property Analysis

**Figure 4:**
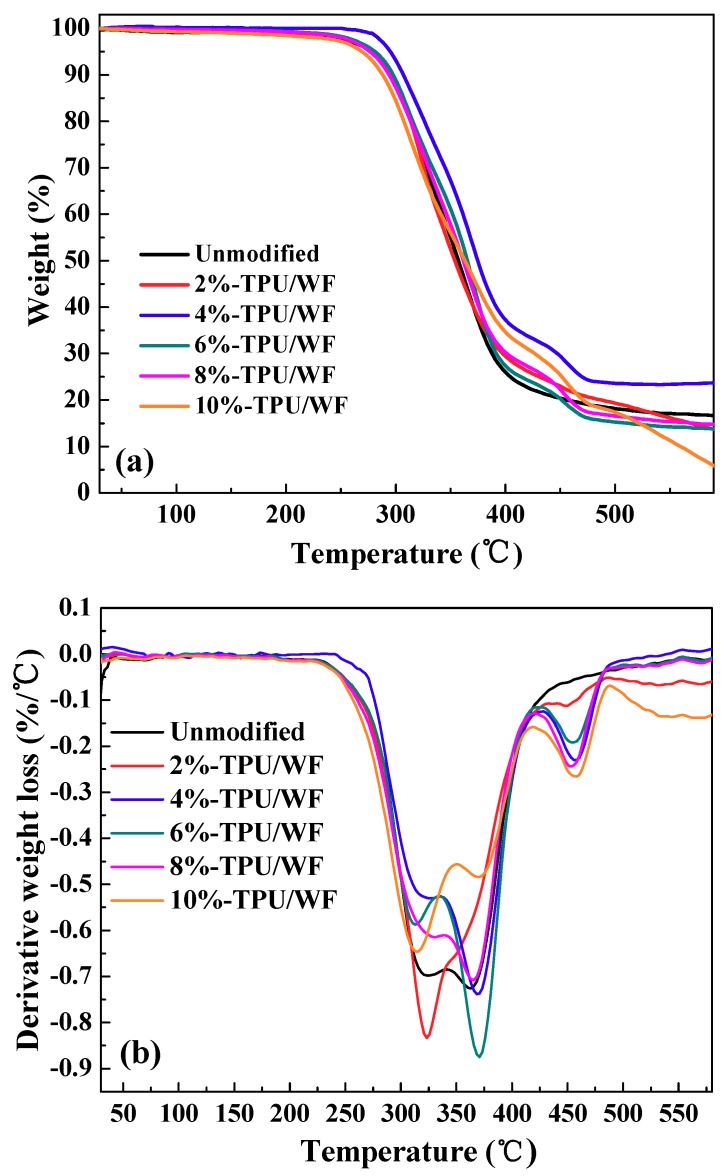
TGA (**a**) and DTG (**b**) curves of the TPU/WF composites with different contents of EPDM-*g*-MAH.

The thermal stability of the unmodified and modified x%-TPU/WF series composites was investigated by TGA. The TGA and DTG curves of TPU/WF composites are presented in Figure 4. The unmodified-TPU/WF composites showed a two-degradation stage, as clearly indicated by the two DTG peaks (black line in Figure 4b); however, all the TPU/WF composites modified with different contents of EPDM-*g*-MAH presented a three-stage decomposition process. It can be inferred that the third decomposition stage (417–486 °C) was due to the addition of EPDM-*g*-MAH. The first and second degradation stages mainly correspond to the degradation temperature of WF and TPU, respectively [31,32,33].

Weight loss curves (Figure 4a) revealed that the initial degradation temperature of 4%-TPU/WF composites (about 282 °C) was significantly higher than the other composites (about 20 °C higher). In other words, the first degradation temperature of TPU/WF composites can be significantly improved by the addition of EPDM-*g*-MAH. It can be concluded that the thermal stability of 4%-TPU/WF composites was increased compared to the unmodified-TPU/WF composites. The improvement in the thermal stability of 4%-TPU/WF composites due to the suitable content of EPDM-*g*-MAH promoted the interfacial interactions between WF and TPU [31]. This was consistent with the results of the above mentioned properties.

**Figure 5:**
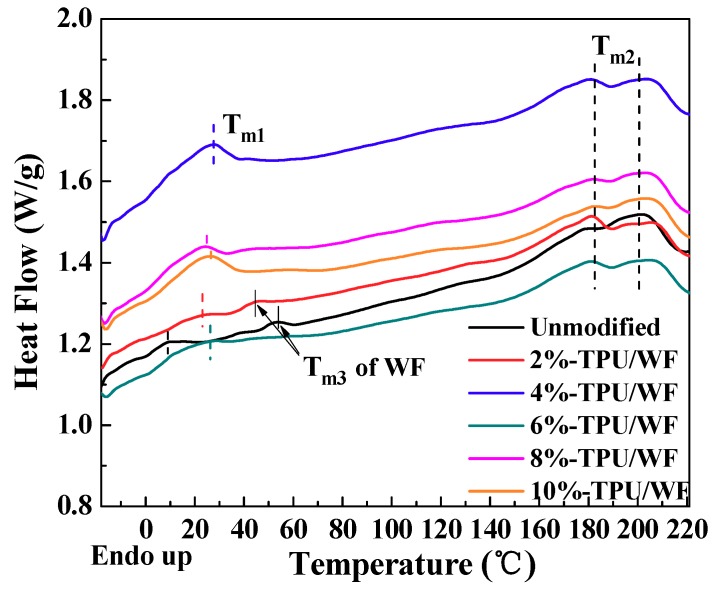
DSC curves of the TPU/WF composites with different contents of EPDM-*g*-MAH.

The DSC second thermograms of unmodified-TPU/WF, 2%-TPU/WF, 4%-TPU/WF, 6%-TPU/WF, 8%-TPU/WF, and 10%-TPU/WF are presented in Figure 5. Figure 5 shows three endothermic peaks at around 15–40 °C (*T*_m1_), 170–190 °C (*T*_m2_), and 50–60 °C (*T*_m3_), which corresponded to the melting temperature of the soft segments, hard segments, and WF, respectively [12,34,35]. It can be seen from the DSC thermogram that the un modified composites had three endothermic peaks, which was mainly because the compatibility between WF and TPU was poor, so the melting peaks of soft segments and WF were relatively obvious. As can be seen from the curve of the 2%-TPU /WF composites, the melting peak (*T*_m1_) of soft segments shifted to the right and the melting peak (*T*_m3_) of WF shifted slightly to the left. This may be because when the EPDM-*g*-MAH content was 2%, it was incompletely contact with WF particles, resulting in a small increase in compatibility. When the content of EPDM-*g*-MAH was 4%, 6%, 8%, and 10%, the composites had two main obvious melting peaks, i.e., *T*_m1_ and *T*_m2_. The results indicated that the addition of EPDM-*g*-MAH significantly improved the compatibility of the composites. The *T*_m1_ of soft segments was often used as the program temperature to actuate the shape memory property of shape memory composites [4,36]. In this study, the program temperature of the shape memory test was set to 60 °C, which was higher than *T*_m1_ of the prepared shape memory TPU/WF composites.

### 3.5. Shape Memory Properties of TPU/WF Composites

**Figure 6:**
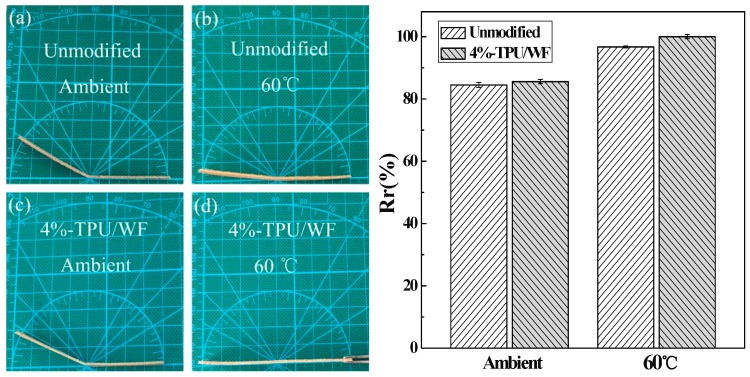
The images of the final changing state (**left**): the unmodified-TPU/WF in the ambient temperature (**a**); the 4%-TPU/WF composites in the ambient temperature (**b**); the unmodified composites at 60 °C (**c**); the 4%-TPU/WF composites at 60 °C (**d**); and the Rr of different TPU/WF composites under different conditions (**right**).

In order to ensure a comparable evaluation of the heat-induced shape memory effect, each group contained two types of specimens (Unmodified and 4%-TPU/WF), while each type was tested at 60 °C in an oven or in the ambient temperature at the same time, respectively. The Rr of the composites under two conditions and the images of the final changing state of the composites at 120 min are shown in Figure 6. It can be seen in Figure 6 that the *R*_r_ of both the unmodified and 4%-TPU/WF composites at 60 °C was higher than the *R*_r_ at ambient temperature. The observation confirmed that the temperature was a critical factor for the heat-induced SME. When the program temperature was 60 °C, it promoted the shape memory performance of TPU/WF composites. In addition, it was found that the *R*_r_ of 4%-TPU/WF composites was slightly higher than the *R*_r_ of unmodified-TPU/WF composites, which indicated that the 4%-TPU/WF composites had better shape memory behavior. The results were consistent with the tensile test results.

**Figure 7:**
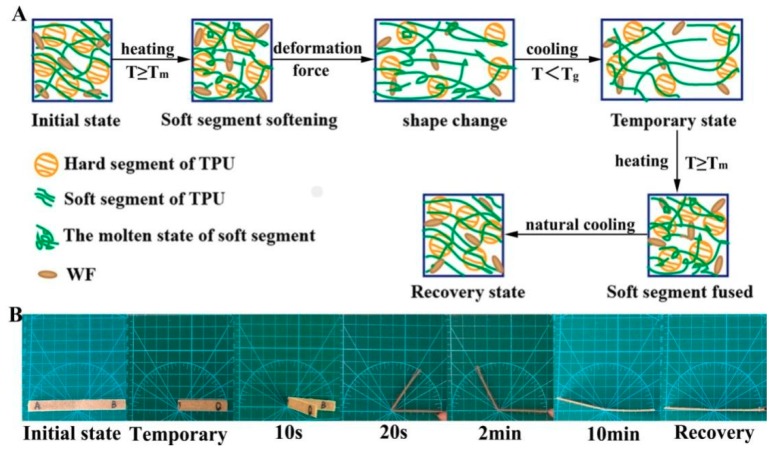
The schematic of the heat-induced shape memory effect mechanism of the TPU/WF composites (**A**) and the shape memory effect of the 4%-TPU/WF composites at 60 °C (**B**).

The schematic of the heat-induced shape memory effect mechanism of the TPU/WF composites and the shape memory effect of the 4%-TPU/WF composites at 60 °C are shown in Figure 7. When the soft segment of TPU was at a high temperature, the molecular chain was extended and orientated under external forces; as a result, they could be deformed to any shapes. When it was cooled below the *T*_g_ of the soft segment, these molecules were in a glassy state and maintained the temporary shape [6,9]. After removing the external forces, the deformed shape would retain the temporary state [9]. Once the temperature was over the *T*_g_ again, the soft segment began to partially melt and the specimen recovered slowly due to conformation hysteresis [9,37,38]. When it was heated to a temperature above *T*_m_ of the soft segment, the soft segment melted, and the deformed structures could return to their original printed shapes automatically. In addition, the effects of the hard segments of TPU and WF were seen on the modulus and strength of the composites at high temperature, so that the composites were deformed in the range above the melted point of the soft segments and below the melted point of the hard segments, without causing fracture or plastic deformation [2].

**Figure 8:**
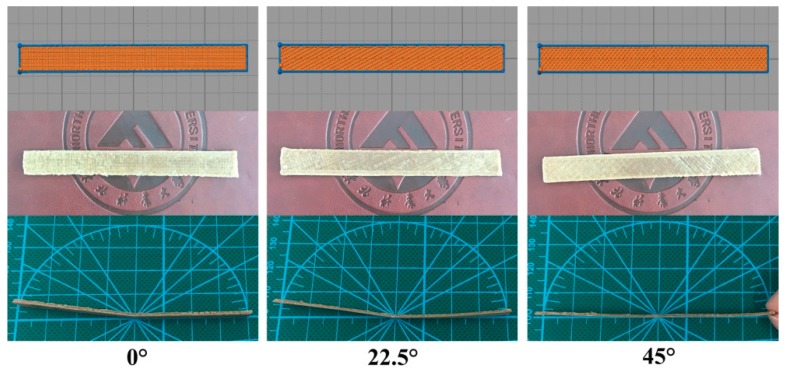
Samples printed from different filling angles and final shape changes of samples after the shape memory performance test.

In order to observe the influence of the filling angle on the shape memory performance of the specimen, the different filling angles of the specimen and the final change state of the specimen after the shape memory performance test were recorded, as shown in the Figure 8. It can be seen in Figure 8 that the final recovery angle of the sample with 45° was greater than 22.5° and 0°. It may be due to the maximum recovery stress of the inclined surface of the specimen at 45° in the recovery process [39]. Similarly, when the filling angle was 22.5°, the recovery force of the specimen was greater than 0°.

### 3.6. Demonstrative Object

**Figure 9:**
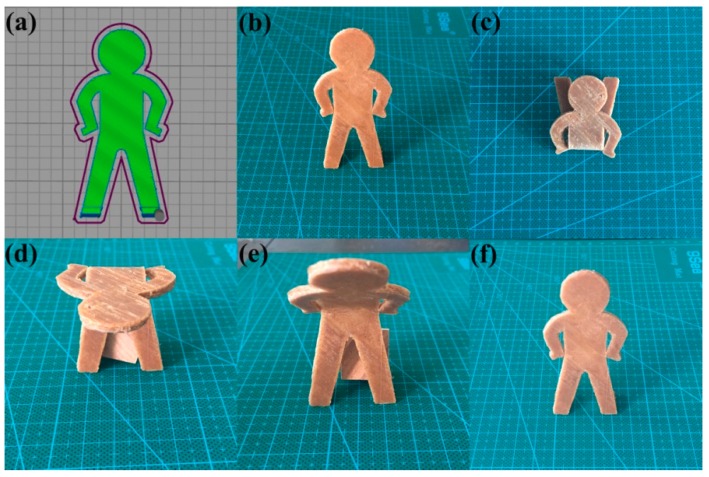
Heat-induced shape memory effect of a man model: the design of the man model (**a**); *S*_o_ (**b**), *S*_t_ (**c**); the recovery state of the model at 2 min (**d**) and 20 min (**e**); *S*_r_ (**f**) at 50 min.

Figure 9 shows the instantaneous shapes of a man model during shape recovery. The object was fabricated from the TPU/WF composite comprising 4% of EPDM-*g*-MAH with the 3D printer, in which the filling angle was 45°. During the recovery in the oven, from the shape *S*_t_ to shape *S*_r_, it can be seen that, quickly after heating, the object moved autonomously, gradually returning to its original shape.

## 4. Conclusions

In this work, a series of heat-induced shape memory TPU/WF composites with different contents of EPDM-*g*-MAH were prepared through hot-melt extrusion and 3D printing. The introduction of EPDM-*g*-MAH can improve the compatibility of TPU and WF. The change of EPDM-*g*-MAH content resulted in different tensile properties of TPU/WF composites. The tensile test results showed that both the tear elongation and tensile strength of the composites firstly increased and then decreased as the EPDM-*g*-MAH content increased. The experimental results showed that, when the EPDM-*g*-MAH content was 4%, the WF could disperse well in the composites, and strong interfacial bonding between WF and TPU was achieved, which can highly improve the composites’ tear elongation without compromising the strength. At the same time, SEM, DMA, TGA, and DSC also indicated that the interface compatibility of the composites was best when the addition of EPDM-*g*-MAH was 4%. In addition, the 4%-TPU/WF composites had a better recovery ratio than unmodified-TPU/WF composites. The shape memory property tests suggested that the temperature was important for affecting the heat-induced shape memory, as well as the filling angle of the specimen. Furthermore, the results of this work can provide useful guidelines for researchers. It is expected that the more interesting behaviors of heat-induced biomass composites and other more challengeable actuation functions, such as soft robotics, sensing, smart products, flexible electronics, and four-dimensional (4D) printing, will be developed [2,9,22].

## Figures and Tables

**Table 1:** The printing parameters used for the FDM printer of EPDM/TPU/WF composites.

Parameter	Value
Nozzle size (mm)	1
Layer thickness (mm)	0.2
Infill density (%)	100
Printing speed (mm/s)	25
Nozzle temperature (°C)	230
Buildplate temperature (°C)	30
Filing structure	rectilinear
Filling angle (°)	45
